# Prioritizing fodder species based on traditional knowledge: a case study of mithun (*Bos frontalis*) in Dulongjiang area, Yunnan Province, Southwest China

**DOI:** 10.1186/s13002-017-0153-z

**Published:** 2017-05-04

**Authors:** Yanfei Geng, Guoxiong Hu, Sailesh Ranjitkar, Yuhua Wang, Dengpan Bu, Shengji Pei, Xiaokun Ou, Yang Lu, Xuelan Ma, Jianchu Xu

**Affiliations:** 1 Institute of Ecology and Geobotany, Yunnan University, Kunming, 650091 China; 2 Key Laboratory of Economic Plants and Biotechnology, Kunming Institute of Botany, Chinese Academy of Sciences, Kunming, 650201 China; 3 University of Chinese Academy of Sciences, Beijing, 100049 China; 4 CAAS-ICRAF Joint Lab on Agroforestry and Sustainable Animal Husbandry, World Agroforestry Centre East and Central Asia, Beijing, 100193 China; 5 State Key Laboratory of Animal Nutrition, Institute of Animal Science, Chinese Academy of Agricultural Sciences, Beijing, 100193 China; 6 College of Life Sciences, Guizhou University, Guiyang, 550025 China; 7 World Agroforestry Centre East and Central Asia, Kunming, 650201 China; 8 Hunan Co-Innovation Center of Animal Production Safety, CICAPS, Changsha, 410128 China

**Keywords:** Traditional knowledge, Dulong people, Wild fodder plants, Tree fodder, Mithun

## Abstract

**Background:**

Livestock rearing is one of the oldest and most important types of smallholder farming worldwide. The sustainability of livestock production depends on the efficient utilization of locally available resources. Some traditional methods of raising livestock may offer valuable lessons in this regard. This study documented and evaluated local knowledge of wild forage plants in the Dulongjiang area in Southwest China in the context of rearing mithun (*Bos frontalis*) in order to provide a sound evidence base for tree fodder selection and the establishment of integrated tree-crop-livestock systems.

**Methods:**

The snowball technique was used to identify key informants with specific knowledge about the topic. Free listing and semi-structured interviews were conducted with 58 households. Participatory investigation and transit walks were used to investigate potential fodder species. Ethnobotanical information was collected, documented and organized.

**Results:**

Overall, 142 wild forage plants from 58 families and 117 genera were identified. Species of the Poaceae, Rosaceae and Urticaceae families were most abundant, with 16, 14 and 11 species respectively identified as fodder plants. Our results indicated that tree/shrub forage plays a major role in the diet of mithun, unlike that of other ruminants. Mithun prefers to browse and move around the forest in search of food, particularly rough and even barbed leaves. Tree species like *Debregeasia orientalis*, *Saurauia polyneura* and *Rubus* species were identified as being important fodder sources. Farmers in this area have traditionally relied on common property resources such as community-managed forests and grasslands to feed their livestock. Farmers have strong incentive to raise mithuns rather than other livestock species due to Dulong people’s cultural preferences.

**Conclusions:**

The wide variety of plants cited by the informants demonstrate the importance of traditional knowledge in gathering information about forage resources. This diversity also offers the prospect of identifying promising species which could be used as fodder plants. Identifying such species and tree fodder species in particular could help smallholder farmers to integrate trees, livestock and crops as part of a sustainable farming system.

## Background

Many smallholder farming methods around the world integrate livestock and crop production. Such integration provides draught power for land management and manure for maintaining cropland fertility. In addition, livestock are a critical source of nutrition. China’s consumption of meat, and particularly of pork, has increased tremendously as its economy has grown. 50 to 80% of all pigs produced in China originate from smallholder farms [1, 2]. China’s rapid economic development and lifestyle transformation have resulted in a growing demand for livestock, and as a result massive restructuring of the livestock sector is underway. New policies and trade agreements have liberalized and industrialized Chinese agriculture, which has enabled enormous increases in production. However, many smallholder farms, which remain a vital part of Chinese rural and indigenous communities, are struggling to survive in the new market-oriented agro-economy [3, 4].

Although economically vulnerable, smallholder systems which integrate crops and livestock continue to be a vital part of agricultural production in China [1, 5]. Smallholder farmers rely on their observations and experience in feeding and managing their livestock. The sustainable production of livestock usually involves efficient utilisation of locally available resources, particularly feed resources. Understanding the importance of wild forage plants is essential for the efficient utilisation of available forest resources [6, 7]. Exploring the potential for growing such resources in intercropping systems with crops and livestock could help smallholder farmers effectively use their limited land resources. Such integration could mitigate the increasing pressure on land and forest resources generated by growing demand for expansion of agricultural and grazing land [8, 9].

Compared to forage grass, tree fodder is particularly important in providing livestock with nutritious food during the dry season when other feed sources are in limited supply [10]. Documenting fodder plants and promoting suitable types of tree fodder for use in home gardens or croplands could be an efficient way to improve sustainable livestock rearing and to change the free grazing style to stall feeding in the mountain without damaging the environment. A combination of traditional and scientific knowledge has been shown to optimize the selection of useful fodder plants [11].

Indigenous people with long histories of livestock rearing may have acquired valuable stores of knowledge about potential fodder/forage resources. Traditional knowledge of fodder plants has been documented in studies of different indigenous groups in several countries, such as Ethiopia, Uganda, Nigeria, India, and Mexico [11–13]. In China, there is an urgent need to document farmers’ knowledge of fodder plants and apply it to the design of tree-crop-livestock systems which could improve smallholder farming [14, 15]. Therefore, this study aims to document local knowledge regarding utilisation and selection of wild forage plants based on indigenous knowledge. The results of this study could be used to provide a shortlist of fodder/forage resources for further nutritional investigation and possible promotion.

## Methods

### Study area

**Fig. 1:**
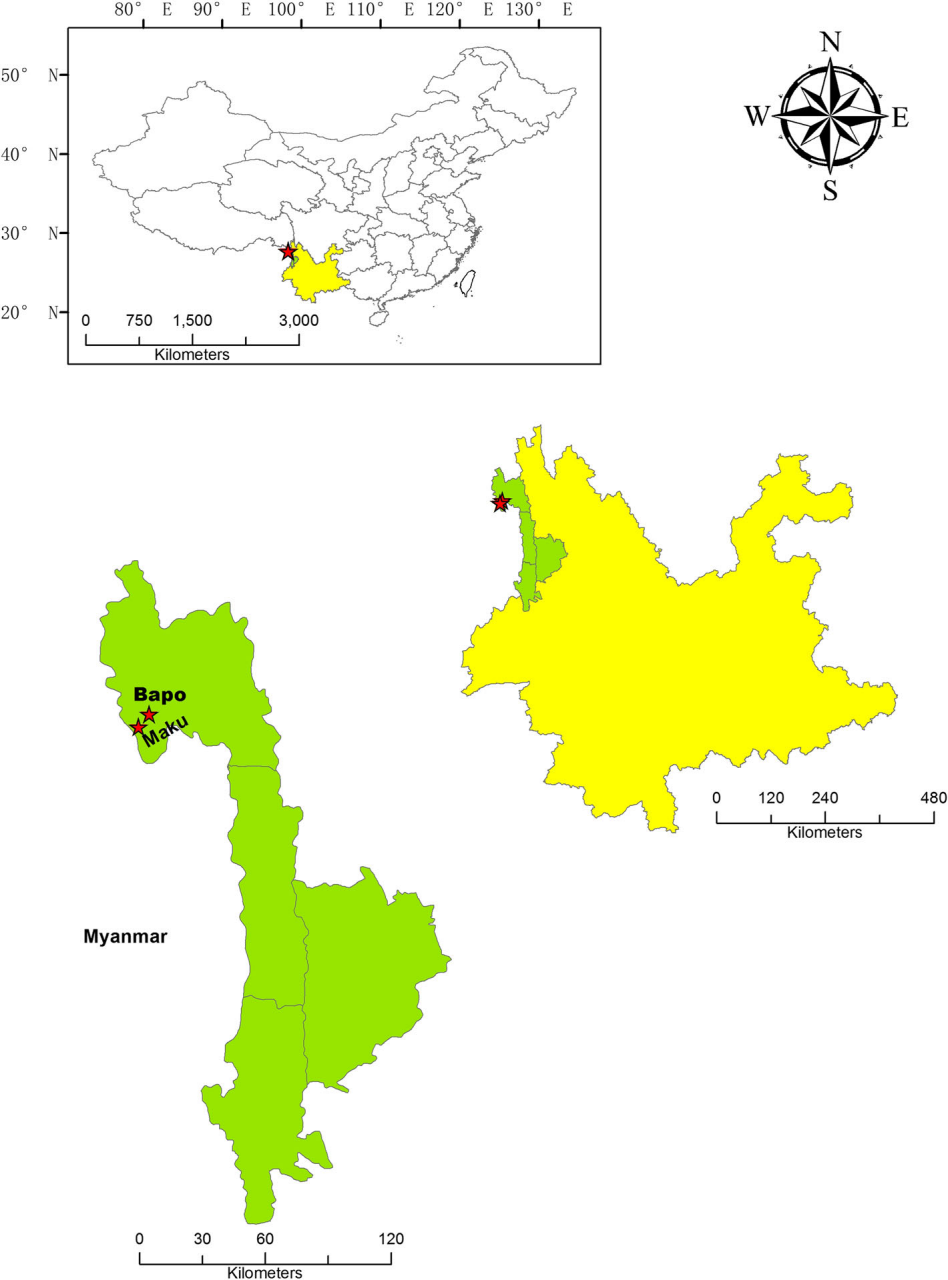
The study area in Dulongjiang, Gongshan County, Yunnan Province, China

**Fig. 2:**
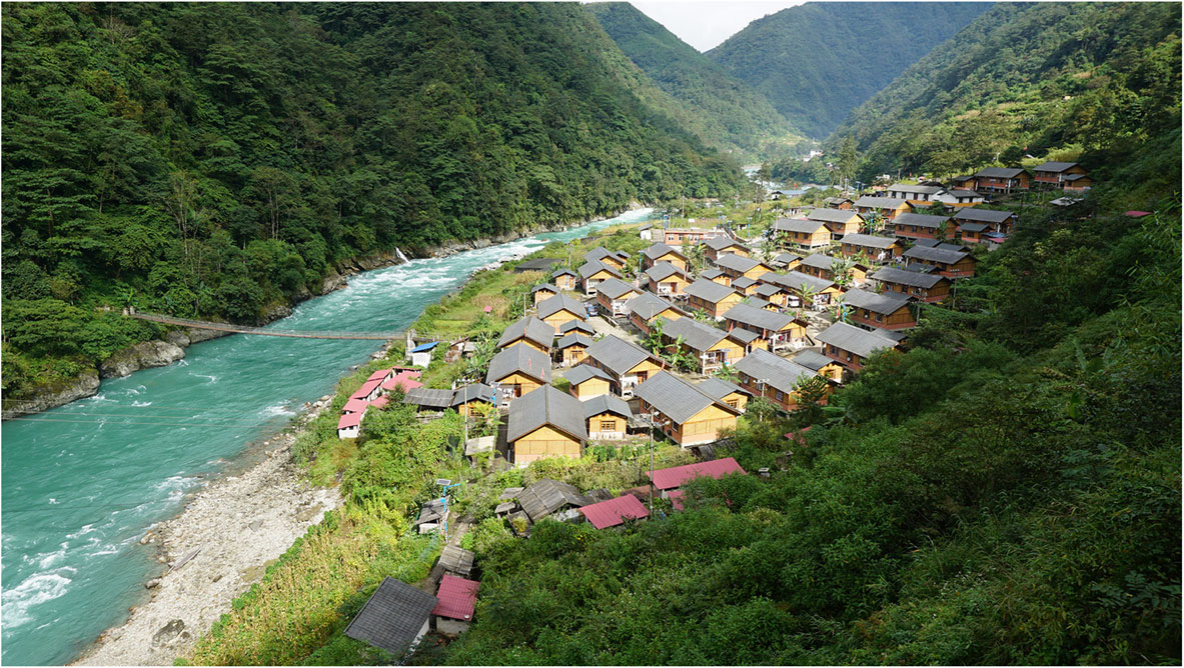
The landscape of study area

The Dulong ethnic group, also known as Drung, is one of the smallest ethnic groups in China and number only 7000 people. Four thousand of them live in Dulongjiang Township, Gongshan County, Yunnan Province, China (Fig. 1). This township (27°44'9"N, 98°20'59"E) was the last town in China to be linked by road (Fig. 2). Before 1956, the Dulong people led a relatively isolated life. Slash-and-burn cultivation was practiced on the local mountains until the 1990s, when it was banned by the government. The Dulong language has no script, and the Dulong traditionally made records and transmitted message by means of engraving notches in wood and tying knots [16]. This lack of written records means that local traditional knowledge is particularly vulnerable to being lost and forgotten. There is therefore an urgent need to document the indigenous knowledge associated with this community.

The study area is a typical alpine-gorge area, ranging from an altitude of 3000 to 4000 meters with almost 85% of the area on steep slopes of more than 25 degrees. The average rainfall is 3672.8 mm per annum [17]. The area contains 4000 species of higher plants belonging to 280 families and 1068 genera. The Dulong people have an expansive knowledge of the diversity and nutritional potential of the local plants which they use for various purposes. According to elder informants, they have been raising mithun (*Bos frontalis*) for more than a hundred years. Mithun is a rare semi-domesticated bovine species which is raised mountain areas, mainly for meat, and is distributed only in India, China, Myanmar, Bhutan, Malaysia and Bangladesh [18]. Mithun plays an important role in the economic, social and cultural life of Dulong people. The meat of mithun raised in the traditional way is high in protein (19.56%) and low in fat (0.36%) [19]. However, this species is vulnerable to extinction according to the International Union for Conservation of Nature and Natural Resources (IUCN) [20].

China’s Tui Geng Huan Lin or “Returning Farmland to Forest” (RFFP) program is the world’s largest and most successful payment for ecosystem services program. This program is a major contributor to China’s dramatic increases in forest cover from perhaps as low as 8% in 1960 to about 21% in 2013 [21]. In Dulongjiang area, RFFP compensated rural households for the conversion of upland farmland to forestland and financed the afforestation of barren mountainsides. Traditionally, local farmers would employ slash-and-burn cultivation to clear land for grass which would provide the mithun with a fast-growing food source. However, after the government banned slash-and-burn methods in 1990s, the expansion of forests has resulted in a lack of available grassland, which poses a threat to the continued raising of mithun. The use of fodder trees could provide a solution to this problem, but currently inadequate information is available on the forage plants consumed by mithun.

In the present study, we 1) accessed farmers’ knowledge of potential forage plants for mithun; 2) documented some potentially useful fodder trees/shrubs; and 3) prioritized important species based on indigenous knowledge. Within this context, the objective of this study is the identification, selection and evaluation of herbaceous and tree species in Dulongjiang area as sources of fodder for mithun.

### Data collection

Our field work revealed that mithun is commonly raised only in Maku(马库), Bapo(巴坡) and Kongdang(孔当) villages, and that there are also a few mithuns in three other villages in the Dulongjiang area. The first two villages are near to Myanmar, which according to our informants is the origin of the local mithun. Our fieldwork was conducted accordingly in Bapo(巴坡) and Maku(马库) villages (Fig. 1) in 2015 and 2016. Field studies included free lists, semi-structured interviews and participatory investigation. A total of 58 key informants were selected using snowball sampling. The ages of informants ranged from 23 to 71, and the mean age was 45 years old. Because activities related to mithun are generally performed by men, we interviewed only two female informants.

In the first stage of the field research, participants were invited to free list all wild fodder plants favored by mithun. The interviews included the following questions: (1) What plants do mithun eat? (2) What are the mithun’s favourite grass and tree fodder sources? (3) What makes mithun grow fast? (4) During the mithun’s calving and nursing periods, what plants do they prefer? (5) What habitats do mithun prefer? (6) What are the feeding habits of mithun? (7) What are the main threats to the mithun population? (8) How can we conserve the present population?

In the second stage of the field research, researchers made a transit walk accompanied by local villagers to collect fodder plants and observe mithun browsing in their natural habitat. Nomenclature of all vascular plants follows Flora of China, and the voucher specimens were deposited at the herbarium of the Kunming Institute of Botany, CAS (KUN).

### Data analysis

Ethnobotanical information collected from 58 key informants was documented and organized. Potential fodder species were prioritized according to the consensus reached by informants. Citation frequency (how many times each plant was mentioned by the informants) was used as a measure of consensus. The frequency of citation was the key factor in prioritizing the fodder species, while other ethnobotanical information such as medicinal function, palatability and availability were also considered. Relevant graphical presentations of the documented plants were prepared. Furthermore, the results of our field investigation of fodder plants for mithun were compared with plant lists in the relevant published literature to understand the diet of mithun fully.

## Results and discussion

### Traditional knowledge of wild fodder plants among Dulong people

**Fig. 3:**
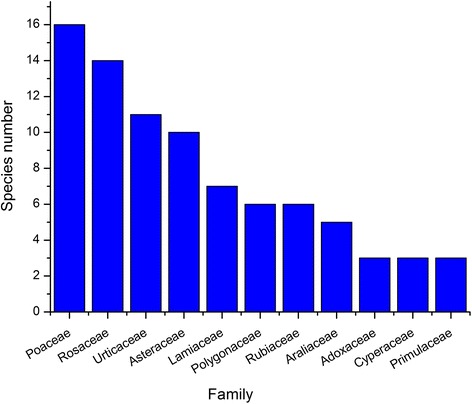
Frequently cited families of wild fodder plant species

Our study revealed that Dulongjiang mithun fed on a highly diverse range of plants. The 58 informants told the interviewers about 142 wild forage plants (Table 1) which belong to 58 families and 117 genera, including 61 species of tree/shrub fodder plants and 81 species of herb forage plants. The arrangement of Angiosperm families is in line with the Angiosperm Phylogeny Website, version 13 (http://www.mobot.org/MOBOT/Research/APweb/welcome.html), and that of ferns follows Christenhusz et al. [22]. Species of the Poaceae, Rosaceae and Urticaceae family were cited most often, with 16, 14 and 11 species respectively (Fig. 3). The wild fodder plants of the Poaceae family included not only various herbs but also tree fodder plants. These were mostly bamboos, and the genus *Gaoligongshania*, a bamboo, is endemic to this mountain area [23]. Forage plants of the Rosaceae and Urticaceae families were often shrubs and herbs respectively. Shrub forage plants of the Rosaceae family were mostly from the genus *Rubus*. Forage plants from the Urticaceae family were mostly herbs. In addition to these three families, respondents also mentioned many wild forage plants from the Asteraceae, Lamiaceae, Polygonaceae and Rubiaceae families, indicating a high local diversity of fodder plants.

**Table 1:** Fodder species consumed by mithun *(Bos frontalis)* in Dulongjiang area, Yunnan Province, Southwest China

Family	Scientific name	Vernacular name	Parts consumed	Multifunctional fodder	Life form	Mithun Preference	Abundance	Voucher no.
Athyriaceae	*Diplazium viridissimum* Christ		whole plant	no	Herb	*	**	G0026
Blechnaceae	*Woodwardia biserrata* C. Presl	risonglabo	leaves	no	Herb	**	**	G0061
Dryopteridaceae	*Dryopteris wallichiana* (Sprengel) Hylander	resang	whole plant	no	Herb	**	**	GYF9
Gleicheniaceae	*Diplopterygium giganteum* (Wallich ex Hooker & Bauer) Nakai		leaves	no	Herb	*	****	G0002
Plagiogyriaceae	*Plagiogyria virescens* (C. Chr.) Ching	mingwa	whole plant	no	Herb	*	*	G0003
Polypodiaceae	*Arthromeris nigropaleacea* S. G. Lu	miwaxin	whole plant	no	Herb	**	*	G0058
Polypodiaceae	*Lepisorus scolopendrium* (Ham.ex D.Don.) Menhra ex Bir		whole plant	no	Herb	**	**	GYF55
Pteridaceae	*Coniogramme caudata* (Wall.) Ching	dayexin, wanjiegelang	leaves	no	Herb	**	**	GYF6
Actinidiaceae	*Saurauia polyneura* C. F. Liang & Y. S. Wang	damujiu	tender branches, leaves	yes, fruits for people	Tree	****	****	GYF41
Adoxaceae	*Sambucus williamsii* Hance		leaves	yes, medicinal plants	Tree or shrub	**	**	PHO9
Adoxaceae	*Viburnum cylindricum* Buch.-Ham. ex D. Don	bulu	leaves	yes, huger food for people	Tree or shrub	**	***	GYF43
Adoxaceae	*Viburnum pyramidatum* Rehder		leaves	no	Tree or shrub	*	*	PHO10
Amaranthaceae	*Achyranthes aspera* L.	gula	whole plant	yes, pig’s favorite	Herb	**	*	G0008
Amaranthaceae	*Deeringia amaranthoides* (Lam.) Merr.		leaves, fruits	yes, medicinal plants	Shrub	*	**	PHO1
Anacardiaceae	*Dobinea vulgaris* Buch.-Ham. ex D. Don		leaves	no	Tree	*	**	PHO41
Apiaceae	*Heracleum candicans* Wallich ex de Candolle	bengduowang	whole plant	no	Herb	**	**	PHO53
Apocynaceae	*Periploca calophylla* (Baill.) Roberty	a ren	tender branches, leaves	yes, medicinal plants	Shrub	**	***	GYF8
Araceae	*Arisaema decipiens* Schott	donghe	leaves	yes, huger food for people	Herb	*	**	PHO2
Araceae	*Arisaema* sp.	langdeng	leaves	no	Herb	**	*	PHO3
Araliaceae	*Aralia chinensis* L.	bang a	leaves	yes, huger food for people	Tree	***	*	G0029
Araliaceae	*Brassaiopsis chengkangensis* Hu	nalangxin	leaves	no	Tree	*	***	PHO4
Araliaceae	*Brassaiopsis glomerulata* (Blume) Regel	nalongxin	leaves	no	Tree or shrub	*	***	PHO5
Araliaceae	*Schefflera chinensis* (Dunn) H. L. Li	lengdemg	leaves	no	Tree	**	**	PHO6
Araliaceae	*Trevesia palmata* (Roxb. ex Lindl.) Vis.	lajia	leaves	yes, medicinal plants	Tree	**	***	G0049
Asteraceae	*Artemisia sieversiana* Ehrh. ex Willd.	debulu	leaves	yes, also fodder for cattle	Herb	**	**	GYF38
Asteraceae	*Blumea densiflora* DC.		leaves	no	Shrub	*	****	GYF48
Asteraceae	*Cirsium* sp.	lajian	whole plant	no	Herb	*	**	G0022
Asteraceae	*Crassocephalum crepidioides* (Benth.) S. Moore		whole plant	yes, huger food for people	Herb	**	**	PHO12
Asteraceae	*Himalaiella deltoidea* (DC.) Raab-Straube		whole plant	no	Herb	**	**	GYF53
Asteraceae	*Myriactis nepalensis* Less.	waguigang	whole plant	no	Herb	**	**	G0052
Asteraceae	*Notoseris yakoensis* (Jeffrey) N. Kilian		whole plant	no	Herb	**	***	GYF49
Asteraceae	*Pseudognaphalium affine* (D. Don) Anderb.		whole plant	no	Herb	**	*	PHO13
Asteraceae	*Senecio scandens* Buch.-Ham. ex D. Don		whole plant	no	Herb	**	***	PHO14
Asteraceae	*Sonchus wightianus* Candolle	nu a bulai	whole plant	no	Herb	**	*	PHO15
Balsaminaceae	*Impatiens holocentra* Handel-Mazzetti	zhuiguli	whole plant	no	Herb	**	**	G0020
Betulaceae	*Alnus nepalensis* D. Don		leaves, fruits	no	Tree	*	*****	PHO7
Betulaceae	*Betula alnoides* Buch.-Ham. ex D. Don	dengpurui	leaves	no	Tree	*	**	PHO8
Brassicaceae	*Cardamine flexuosa* With.		whole plant	no	Herb	***	**	PHO17
Caprifoliaceae	*Leycesteria gracilis* (Kurz) Airy Shaw	baguajia	leaves	no	Shrub	**	*	G0048
Caprifoliaceae	*Valeriana barbulata* Diels	dengsen	whole plant	no	Herb	***	**	PHO56
Caryophyllaceae	*Drymaria cordata* (L.) Willd. ex Schult.		whole plant	no	Herb	**	**	PHO11
Caryophyllaceae	*Stellaria vestita* Kurz		whole plant	no	Herb	**	**	GYF37
Celastraceae	*Celastrus gemmatus* Loes.	a ren	leaves	no	Shrub	**	**	GYF1
Commelinaceae	*Commelina paludosa* Blume	bengge	leaves	no	Herb	***	**	GYF33
Coriariaceae	*Coriaria nepalensis* Wall.		leaves	no	Shrub	*	**	PHO16
Cornaceae	*Cornus macrophylla* Wall.	benmiqiang	leaves	no	Tree	**	*	G0051
Cucurbitaceae	*Gynostemma pentaphyllum* (Thunb.) Makino		whole plant	no	Herb	**	**	PHO18
Cyperaceae	*Carex baccans* Nees	jiwoka	whole plant	no	Herb	***	**	GYF51
Cyperaceae	*Carex nubigena* D. Don ex Tilloch & Taylor	jingwo	whole plant	no	Herb	***	***	PHO19
Cyperaceae	*Scleria dulungensis* P. C. Li		whole plant	no	Herb	***	**	PHO20
Ericaceae	*Vaccinium gaultheriifolium* (Griff.) Hook. f. ex C.B. Clarke	kaixin	leaves	no	Shrub	**	*	PHO21
Fabaceae	*Pueraria lobata* (Willd.) Ohwi	buruikale	leaves	yes, huger food for people	Herb	***	**	PHO28
Gentianaceae	*Tripterospermum chinense* (Migo) Harry Sm.		leaves	no	Herb	***	***	GYF46
Gesneriaceae	*Aeschynanthus superbus* C. B. Clarke		leaves	no	Shrub	**	*	PHO22
Hydrangeaceae	*Hydrangea longipes* Franch.	benming	leaves	no	Shrub	***	****	GYF24
Hypericaceae	*Hypericum addingtonii* N. Robson		leaves	no	Shrub	*	**	GYF4
Hypoxidaceae	*Curculigo capitulata* (Lour.) O. Ktze.	xiaowei	whole plant	no	Herb	***	**	GYF40
Hypoxidaceae	*Curculigo sinensis* S. C. Chen	shiwei	whole plant	no	Herb	****	**	G0001
Iteaceae	*Itea kiukiangensis* Huang & S. C. Huang ex H. Chuang		leaves	no	Tree	**	**	PHO23
Lamiaceae	*Ajuga nipponensis* Makino		leaves	no	Herb	**	*	PHO24
Lamiaceae	*Craniotome furcata* (Link) Kuntze	mengsang	whole plant	no	Herb	**	****	GYF20
Lamiaceae	*Elsholtzia blanda* (Benth.) Benth.		tender branches, leaves	no	Herb	**	*	PHO25
Lamiaceae	*Leucosceptrum canum* Sm.	xindong	leaves	no	Tree or shrub	**	****	PHO26
Lamiaceae	*Melissa axillaris* (Benth.) Bakh.f.	renangsangxi	whole plant	no	Herb	**	***	GYF11
Lamiaceae	*Pogostemon brevicorollus* Y. Z. Sun ex C. H. Hu		whole plant	no	Herb	**	****	GYF10
Lamiaceae	*Prunella vulgaris* L.	wagui	whole plant	yes, medicinal plants	Herb	***	**	PHO27
Magnoliaceae	*Magnolia* sp.	sirbeng	leaves	no	Tree	*	*	GYF26
Melanthiaceae	*Paris* sp.	chonglou	leaves	yes, mithun growing fast and medicinal plants	Herb	***	**	PHO29
Melastomataceae	*Oxyspora yunnanensis* H. L. Li		leaves	no	Shrub	**	**	GYF45
Musaceae	*Ensete wilsonii* (Tutcher) Cheesman	gelong	leaves	yes, huger food for people	Herb	**	*	PHO31
Oleaceae	*Jasminum lanceolarium* Roxb.		tender branches, leaves	no	Woody liana	*	*	GYF42
Orchidaceae	*Cymbidium faberi* Rolfe	xinwa	whole plant	no	Herb	*	*	G0059
Orchidaceae	*Pholidota articulata* Lindl.		whole plant	no	Herb	*	*	GYF5
Plantaginaceae	*Plantago asiatica* L.	wagui	whole plant	yes, medicinal plants	Herb	***	**	G0004
Poaceae	*Arundo donax* L.	gelu	tender branches, leaves	yes, mithun growing fast with shiny fur	Tree	****	*****	G0056
Poaceae	*Chimonobambusa armata* (Gamble) Hsueh & T. P. Yi	jiu	leaves, bamboo shoot	yes, huger food for people	Tree	****	****	PHO34
Poaceae	*Dendrocalamus fugongensis* Hsueh & D. Z. Li	duwa	leaves, bamboo shoot	yes, huger food for people	Tree	***	***	PHO35
Poaceae	*Erianthus longisetosus* T. Anderson	shiling	whole plant	yes, mithun growing fast with shiny fur	Herb	*****	*****	G0060
Poaceae	*Fargesia praecipua* T. P. Yi	sameng	leaves, bamboo shoot	yes, huger food for people	Tree	****	****	GYF21
Poaceae	*Gaoligongshania megalothyrsa* (Hand.-Mazz.) D.Z. Li, J.R. Xue & N.H. Xia	langsa	leaves, bamboo shoot	yes, huger food for people	Tree	*	****	GYF22
Poaceae	*Imperata cylindrica* (L.) P. Beauv.	aji	whole plant	yes, mithun growing fast with shiny fur	Herb	****	***	PHO36
Poaceae	*Isachne albens* Trin.	mieqie	whole plant	no	Herb	***	***	GYF35
Poaceae	*Microstegium nudum* (Trin.) A. Camus	yilong	whole plant	no	Herb	***	**	G0013
Poaceae	*Miscanthus nepalensis* (Trin.) L. Liu		whole plant	no	Herb	***	***	PHO8406
Poaceae	*Oplismenus compositus* (L.) P. Beauv.	jilong	whole plant	no	Herb	***	**	GYF36
Poaceae	*Pennisetum alopecuroides* (L.) Spreng.		whole plant	no	Herb	****	**	PHO37
Poaceae	*Phyllostachys mannii* Gamble	remeng	leaves, bamboo shoot	yes, huger food for people	Tree	***	***	PHO38
Poaceae	*Saccharum arundinaceum* Retz.	hong	whole plant	no	Herb	****	***	GYF39
Poaceae	*Sacciolepis indica* (L.) Chase	ji ong	whole plant	no	Herb	**	*	PHO39
Poaceae	*Setaria plicata* (Lam.) T. Cooke		whole plant	no	Herb	***	***	PHO40
Polygonaceae	*Fagopyrum dibotrys* (D. Don) H. Hara	shili	whole plant	yes, fodder for all livestocks huger food for people	Herb	**	*	GYF30
Polygonaceae	*Polygonum capitatum* Buch.-Ham. ex D. Don	longgadebeng	whole plant	no	Herb	**	**	PHO42
Polygonaceae	*Polygonum chinense* var. *hispidum* Hook. f.		whole plant	no	Herb	***	***	GYF34
Polygonaceae	*Polygonum molle* D. Don	yibangge, bengge	tender branches, leaves	yes, huger food for people	Shrub	****	****	G0032
Polygonaceae	*Polygonum runcinatum* Buch.-Ham. ex D. Don	zhebamu	whole plant	yes, huger food for people	Herb	***	**	PHO43
Polygonaceae	*Polygonum runcinatum* var. *sinense* Hemsl.	debeng	whole plant	yes, medicinal plants	Herb	***	***	G0030
Primulaceae	*Embelia floribunda* Wall.		tender branches, leaves	no	Shrub	**	*	PHO32
Primulaceae	*Embelia parviflora* Wall. ex A. DC.		tender branches, leaves	no	Shrub	*	*	GYF27
Primulaceae	*Myrsine semiserrata* Wall.	xinjixin	tender branches, leaves	no	Shrub	**	***	PHO33
Ranunculaceae	*Clematis armandii* Franch.		whole plant	yes, medicinal plants	Herb	***	**	PHO44
Ranunculaceae	*Clematis* sp.		whole plant	yes, medicinal plants	Herb	**	**	GYF14
Rosaceae	*Fragaria moupinensis* (Franch.) Cardot	bohuo	whole plant	yes, fruits for people	Herb	***	**	G0007
Rosaceae	*Fragaria pentaphylla* Losinsk.	buleng	whole plant	yes, fruits for people	Herb	***	**	PHO45
Rosaceae	*Geum aleppicum* Jacq.		whole plant	no	Herb	***	***	PHO46
Rosaceae	*Neillia thrysiflora* D. Don		leaves	no	Tree	**	**	GYF47
Rosaceae	*Potentilla kleiniana* Wight & Arn.	denggabune	whole plant	no	Herb	**	*	PHO47
Rosaceae	*Rubus biflorus* Buch.-Ham. ex Sm.	benheqi	tender branches, leaves, fruits	yes, fruits for people	Shrub	**	**	G0043
Rosaceae	*Rubus ellipticus* Sm.	benghe, bengge	tender branches, leaves, fruits	yes, fruits for people	Shrub	**	**	GYF13
Rosaceae	*Rubus irenaeus* Focke		tender branches, leaves, fruits	yes, fruits for people	Shrub	***	***	GYF25
Rosaceae	*Rubus lineatus* Reinw.	jiawa	tender branches, leaves, fruits	yes, fruits for people	Shrub	****	*****	G0046
Rosaceae	*Rubus mesogaeus* Focke		tender branches, leaves, fruits	yes, fruits for people	Shrub	**	****	PHO48
Rosaceae	*Rubus paniculatus* Sm.	benghe	tender branches, leaves, fruits	yes, fruits for people	Shrub	***	**	G0005
Rosaceae	*Rubus pectinarioides* H. Hara		tender branches, leaves, fruits	yes, fruits for people	Shrub	**	*	GYF23
Rosaceae	*Rubus pentagonus* Wall. ex Focke	dachui	tender branches, leaves, fruits	yes, fruits for people	Shrub	**	*	GYF54
Rosaceae	*Rubus taronensis* C. Y. Wu ex T. T. Yu & L. T. Lu	lajia	tender branches, leaves, fruits	yes, fruits for people	Shrub	**	**	GYF19
Rubiaceae	*Damnacanthus indicus* C. F. Gaertn.		leaves	no	Shrub	*	**	GYF12
Rubiaceae	*Hedyotis scandens* Roxb.		whole plant	no	Herb	*	**	GYF18
Rubiaceae	*Luculia yunnanensis* S. Y. Hu	gudie, xiu	leaves	no	Shrub	**	**	GYF44
Rubiaceae	*Ophiorrhiza lurida* Hook.f.		whole plant	no	Herb	*	**	GYF29
Rubiaceae	*Uncaria scandens* (Sm.) Hutch.		tender branches, leaves	no	Woody liana	*	*	GYF52
Rubiaceae	*Wendlandia speciosa* Cowan		leaves	no	Tree or shrub	*	**	PHO49
Rutaceae	*Toddalia asiatica* (L.) Lam.		tender branches, leaves	no	Shrubs or woody climbers	***	**	PHO50
Sabiaceae	*Sabia dielsii* Levl.		tender branches, leaves	no	Woody liana	*	**	GYF50
Saxifragaceae	*Astilbe chinensis* (Maxim.) Franch. & Sav.		whole plant	no	Herb	**	**	PHO51
Scrophulariaceae	*Buddleja* sp.		leaves	no	Tree	***	**	PHO30
Smilacaceae	*Smilax lunglingensis* F. T. Wang & Tang	aguaidelei	leaves	no	Shrub	***	***	GYF16
Smilacaceae	*Smilax myrtillus* A. DC.		leaves	no	Shrub	*	*	GYF15
Staphyleaceae	*Turpinia macrosperma* Huang		leaves	no	Tree	*	**	PHO52
Urticaceae	*Debregeasia orientalis* C. J. Chen	xinyi	leaves	no	Shrub	****	****	G0038
Urticaceae	*Elatostema hookerianum* Wedd.	kena, shilikangqiang	whole plant	yes, muntjac’s favorite	Herb	***	**	G0009
Urticaceae	*Elatostema laevissimum* W. T. Wang		whole plant	yes, muntjac’s favorite	Herb	***	**	PHO54
Urticaceae	*Elatostema nasutum* Hook.f.	kangqiang	whole plant	yes, muntjac’s favorite	Herb	**	**	GYF2
Urticaceae	*Elatostema platyphyllum* Wedd.	zhemeng	whole plant	no	Subshrubs	*****	*****	GYF31
Urticaceae	*Gonostegia hirta* (Bl.) Miq.	yileng, jielang	whole plant	no	Herb	****	**	G0053
Urticaceae	*Lecanthus pileoides* Chien & C. J. Chen	wangnuoka, wandong	whole plant	no	Herb	****	**	G0012
Urticaceae	*Oreocnide frutescens* (Thunb.) Miq.		leaves	no	Tree	***	***	PHO55
Urticaceae	*Pilea pellionioides* C. J. Chen		whole plant	no	Herb	***	**	GYF3
Urticaceae	*Pilea* sp.	ganji	whole plant	no	Herb	**	***	G0018
Urticaceae	*Urtica ardens* Link	bengluo	whole plant	no	Herb	*	*	GYF17
Vitaceae	*Tetrastigma obtectum* (Wall. ex M.A. Lawson) Planch. ex Franch.	buleng a ren	tender branches, leaves	yes, fruits for people	Herbaceous liana	***	**	GYF28
Zingiberaceae	*Cautleya gracilis* (Sm.) Dandy	gubeng	leaves	no	Herb	*	*	GYF7
Zingiberaceae	*Hedychium sinoaureum* Stapf	gubaomu, gubeng	leaves	no	Herb	**	*	G0014

Species in inventory are ordered from lower to higher plants, and they are arranged firstly by family taxa and then by genus taxa. Vernacular name of wild edibles are written using Chinese pinyin. * in Column Mithun Preference and Abundance represents the preference level or indicated resource amount, and more *, more preferred or more abundant resources

Informants demonstrated a sophisticated knowledge of nutritional characteristics such as nutritional value (fodder plants which enable mithun to grow rapidly), palatability and availability. About 32% of reported fodders were multifunctional, some of which were said to cause a bodyweight gain and shiny fur. Our informants listed *Arundo donax*, *Paris* sp. and *Erianthus longisetosus* as being very important fodder plants. *A. donax* is a perennial herbaceous plant and promising energy plant, which could serve as an alternative to wood from short-rotation forestry [24–26]. 50% of informants claimed that *E. longisetosus* made mithun fur very black and shiny. *E. longisetosus* is very popular among cattle farmers and was most commonly used in dairy cow feeding [27, 28]. *Paris* sp. is a perennial medicinal plant and is a promising candidate for the development of anti-cancer drugs [29–31], and is important forage plant as identified by the informants. Overall, wild forage plants consumed by mithuns were diverse and abundant in the study site. Some studies point out that herbivores have various mechanisms to prevent absorption or reduce the effect of ingested toxins in the wild grazing [32, 33].

**Fig. 4:**
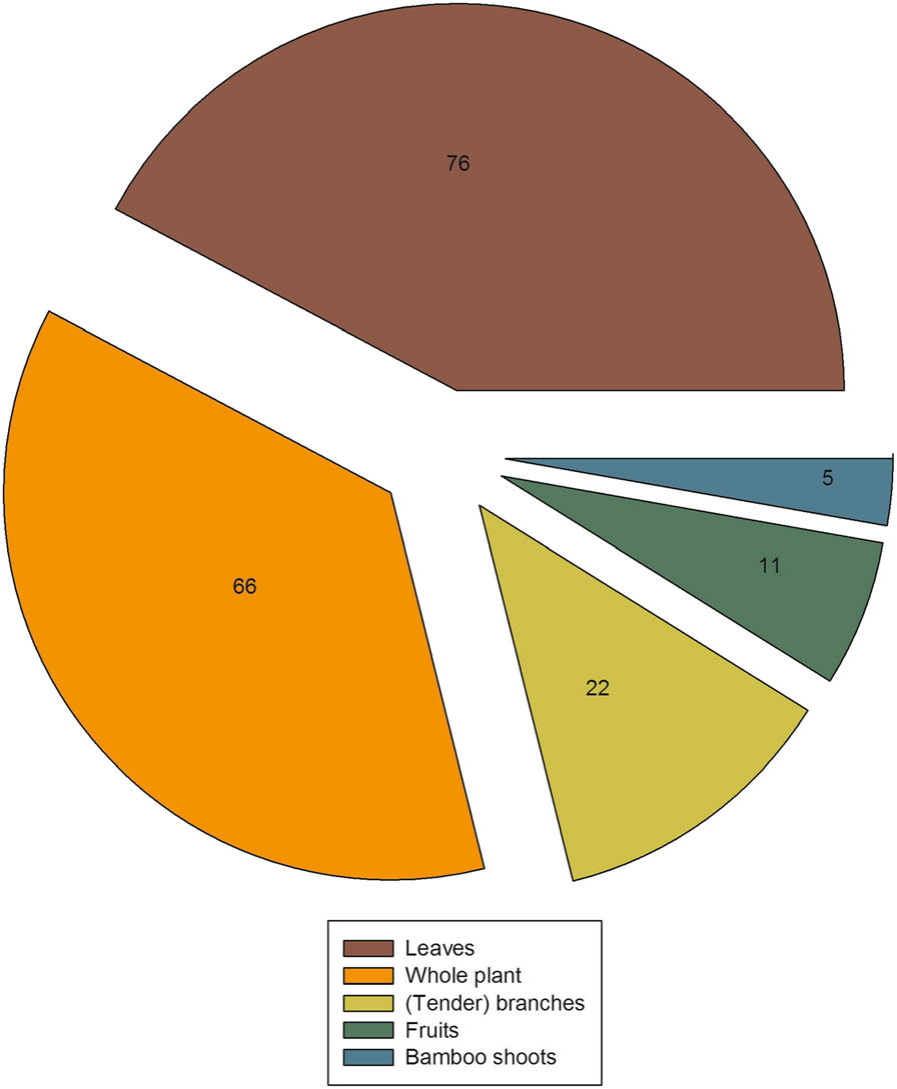
Plant parts consumed and the number of plant parts used

The informants reported that mithun preferentially consumed tender leaves (53.52%). The other parts most frequently consumed were whole plants, tender branches, fruits and bamboo shoots (Fig. 4). Of the plant species cited, 33.8% had only one part of the plant often eaten by mithun, while the remaining species had more than two parts mentioned as animal feed. Due to the climate of the Dulongjiang area, most wild fodder plants documented in the study can also be accessed in the winter, and there was no significant seasonal difference in the plant species consumed by mithun.

For mithun, less consumed fodder species usually were the less distributed species in Dulongjiang area (Table 1), such as *Cymbidium faberi*, *Pholidota articulate* and *Urtica ardens*. But some common fodder species were less consumed by mithuns in Dulongjiang area probably due to their unpleasant taste, such as *Blumea densiflora* and *Alnus nepalensis*.

### Feeding habits, prioritizing fodder trees/shrubs and promotion possibilities

**Fig. 5:**
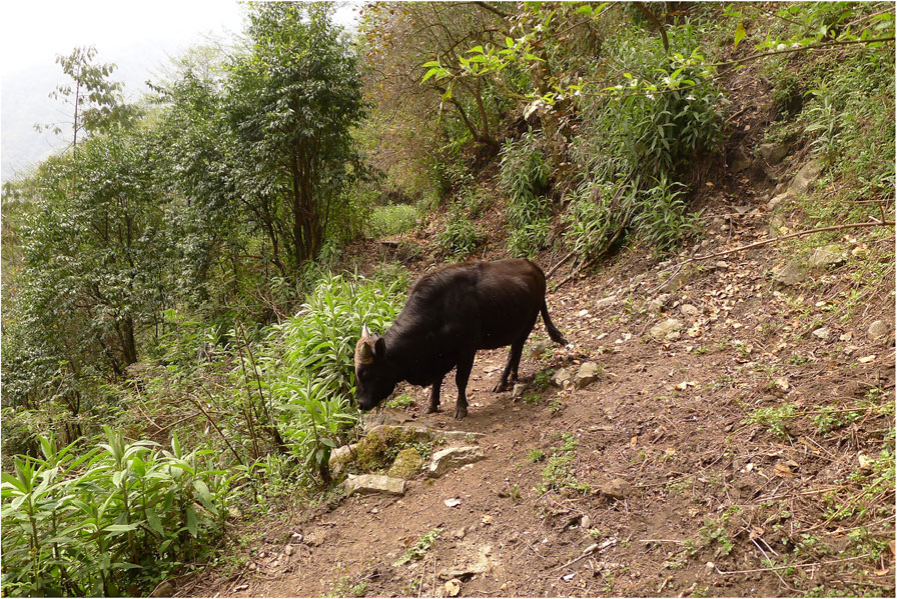
Foraging mithun in the natural habitat

Mithun thrives on jungle forage, tree fodder, shrubs, herbs and other natural vegetation. They stay on the mountain year-round, and farmers do not provide any additional feeding; they merely provide salt three to five times monthly, especially if for some reason it is necessary to restrain the animals from freely ranging around (Fig. 5). Too much salt makes mithun more vulnerable to hypertension, so an adult mithun must be given less than three kilograms of salt per month.

**Fig. 6:**
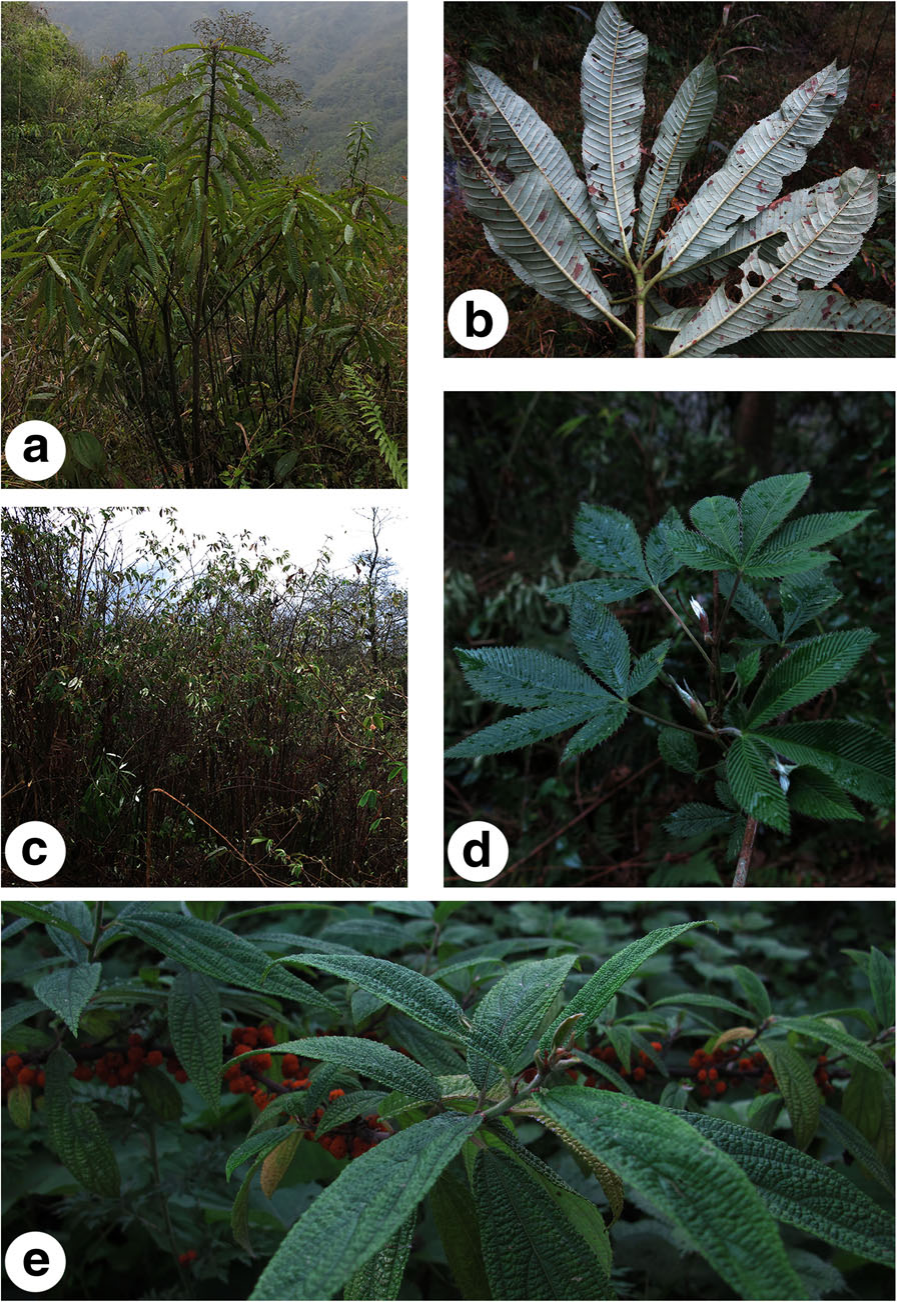
Three preferred fodder tree species: **a**-**b** *Saurauia polyneura*; **c**-**d** *Rubus lineatus*; **e** *Debregeasia orientalis*

Mithun prefers to move around the forest and browse selectively, particularly on rough or even spiny leaves like those of *Debregeasia orientalis*, *Saurauia polyneura* and forage plants in the genus *Rubus*, such as *Rubus lineatus* (Fig. 6). Preferred species are often leafy and without lots of stem, with a high leaf table and leaves of low tensile strength [34]. The species found in the diet of the mithun match with the percentage of species found in the botanical composition of the local forest [35]. According to a European study, the ungulates can play a major role as seed dispersers for plant dispersal in forest habitats [36]. However, comparison of this study’s results with those of Indian mithun reveals no overlap between species consumed by mithun in Dulongjiang and in India. This indicates that mithun can adapt well to local environments in which the availability of different foods is limited during some parts of the year [16, 37].

Tree/shrub fodder plays a major role in the diet of mithun, unlike that of other ruminants. In the Dulongjiang area, tree and herb forage plants mostly belong to the Poaceae family, and shrub fodder plants mostly belong to the Rosaceae family. More than half of the 20 most frequently cited fodder species in our survey (frequency ≥15) were trees or shrubs, and half of them belonged to the Poaceae family (Table 2). *E. longisetosus* and *A. donax* were abundant in the area, growing on riversides and hillsides at lower elevations. At the onset of winter, the mithun moves to lower elevation regions with higher temperatures and more available food, and these two perennial grasses become their important food source. Bamboo is also preferred by mithun but Dulongjiang has relatively low amounts of bamboo. In interviews, *Phyllostachys mannii* was mentioned more frequently than the other two (*Chimonobambusa armata* and *Fargesia praecipua*) (Table 2). This is probably because most of the interviewees seldom go high up into the mountains where the latter two species occur. *Imperata cylindrica* usually occurs in disturbed place, and it became very common in Dulongjiang when both sides of the valley were converted to farmland. However, the recent reforestation program made it less common. In addition to Poaceae, two species of Urticaceae were also mentioned frequently. *D. orientalis* is often found growing next to streams, while *Elatostema hookerianum* grows in limited amounts in the forest. *S. polyneura* and *R. lineatus* are widely distributed throughout the Dulongjiang area, and were used by a smallholder in Gongshan County to feed mithuns in captivity. Local residents also identified species mentioned above as being able to provide high quality woody fodder. Tree fodder plants often have multi-purpose uses. For example, *P. mannii, C. armata* and *F. praecipua* yield edible shoots; *R. lineatus* produces edible fruits and planted as a courtyard ornament [38, 39]; fruits of *Saurauia* have been important edible fruits since hunger time [40, 41].

**Table 2:** Frequently cited fodder species consumed by mithun *(Bos frontalis)* in Dulongjiang area, Yunnan Province, Southwest China

Family	Scientific name	Vernacular name	Cited frequency	Life form
Poaceae	*Erianthus longisetosus* T. Anderson	shiling	47	Herb
Poaceae	*Phyllostachys mannii* Gamble	remeng	37	Tree
Poaceae	*Imperata cylindrica* (L.) P. Beauv.	aji	31	Herb
Urticaceae	*Debregeasia orientalis* C. J. Chen	xinyi	28	Shrub
Poaceae	*Arundo donax* L.	gelu	27	Tree
Urticaceae	*Elatostema hookerianum* Wedd.	kena	24	Herb
Poaceae	*Chimonobambusa armata* (Gamble) Hsueh & T. P. Yi	jiu	20	Tree
Actinidiaceae	*Saurauia polyneura* C. F. Liang & Y. S. Wang	damujiu	20	Tree
Poaceae	*Fargesia praecipua* T. P. Yi	sameng	16	Tree
Rosaceae	*Rubus lineatus* Reinw.	jiawa	15	Shrub
Fabaceae	*Pueraria lobata* (Willd.) Ohwi	buruikale	11	Herb
Poaceae	*Oplismenus compositus* (L.) P. Beauv.	jilong	11	Herb
Polygonaceae	*Polygonum molle* D. Don	bengge	11	Shrub
Polygonaceae	*Fagopyrum dibotrys* (D. Don) H. Hara	shili	9	Herb
Hydrangeaceae	*Hydrangea longipes* Franch.	benming	8	Shrub
Poaceae	*Saccharum arundinaceum* Retz.	hong	8	Herb
Melanthiaceae	*Paris* sp.	chonglou	7	Herb
Poaceae	*Dendrocalamus fugongensis* Hsueh & D. Z. Li	duwa	7	Tree

The use of fodder trees may also bring other benefits to farmers in Dulongjiang. The sale of tree fodder by-products by smallholder farmers provides an opportunity to boost household incomes in the study area. Integrating trees into farming practices often has direct effects on crop production such as weed suppression and pest and disease reduction. Livestock such as mithun may also produce higher returns than single crop production as much of the local area is steeply sloped. The use of fodder trees on cropland could be combined with raising mithun and growing crops in integrated tree-crop-livestock systems which would make efficient use of the available land resources without harming natural forests through agricultural extension.

In many countries, fragmentation of land holdings coupled with the declining productivity of public forests motivates farmers to cultivate more trees on their private farmland in order to develop on-farm tree fodder resources [42–44]. However, one of the disadvantages of growing trees on crop land is a negative impact on crop yield. Some species may be suitable for feeding livestock, but agronomically unsuccessful [45, 46]. Future introductions of fodder plants to new areas and systems may be more successful if based on species and cultivars with long research pedigrees in the same agro-ecologies rather than on unknown species [47], and for which tree-crop interactions have been thoroughly investigated [48].

For many local farming communities, agroforestry is not a new land use practice but a proven production strategy [49]. Our results indicate that this wealth of knowledge can be used to inform the selection of ecologically suitable and economically beneficial fodder trees. These trees could form part of small-scale, mixed tree-crop-livestock systems which could play a major role in improving livelihoods and protecting natural ecosystems in mountain landscapes.

### Mithun farming and livelihoods development

Crop farming, raising livestock and running small businesses were regarded as the most important economic activities in Dulongjiang area. Livestock, especially mithun and pigs, were in the past raised partly for ceremonial purpose but have now become an important source of income. Farmers in this area and the eastern Himalayas have traditionally relied on common property resources such as forest and grasslands to feed their livestock [27, 50]. Currently, Dulongjiang farmers use a joint management system whereby a few families take turns to check the numbers and condition of the mithun, and feed them salt.

When asked about the main threats to the mithun population and means of protecting it, almost all local villagers agreed that feeding mithun in stalls would be a better way to avoid accidental death on the steep hillsides as well as from predators such as bears. However, some worried that feeding mithun in this way could make them weak and thin. Mithun still contribute significantly to the livelihoods of traditional smallholders in Dulongjiang area, and both the introduction of new husbandry practices and the planting of specialized forage plants, even in a limited way, present serious challenges. The sale of mithun could boost income, though the ability to do this is often limited by a lack of capital with which to buy calves . According to some studies, mithuns are not usually raised for their milk, but they become accustomed to milking. Mithun produce around 1–1.5 kg milk/day/animal, and the fat content of their milk varies from 11 to 13% [18, 28]. If mithun could be more readily sold, farmers would have a stronger incentive to keep mithun, in addition to their cultural value and provision of meat [51]. Farmers also prefer to plant economically and commercially valuable trees over fodder trees [52].

The time invested in raising mithun varied widely among individual households. Members of a household spent about five hours per week feeding their mithuns salt and checking on them. Smallholder livestock production also has the advantage of having lower labor costs than either using hired labor on large-scale farms or the cut-and-carry feeding method employed in other regions [53].

Efforts to improve livestock production in smallholder farming systems face various challenges. First, the selection of different species as candidates for planting fodder trees must take account both their ecological suitability and their potential for supporting local livelihoods [54]. Second, farming systems in this area are usually low input, and under high-risk conditions, farmers are reluctant to invest in improved production technologies [55, 56]. However, in the Dulongjiang area, the local government has strongly supported under-forest resource development, and has offered each household *Amomum tsaoko* and *Paris* sp. seedlings for free. Therefore, the local government could play a vital role in establishing an efficient integrated tree-livestock-crop program and convincing farmers that investing in such systems is worthwhile.

The present study on the forage plants known by Dulong people built upon their locally-developed experience during the process of domestication of the mithun. Attention for the conservation of this rare animal is necessary and proper management of forage source is one of the important aspects in the step of conservation. Ethnobotanical experience from one community can be useful for other community. Similarly, practices and policy in one village could be useful for another village. The concept of integrated tree-livestock-crop can be acceptable and applicable in the regions like northeast India and Myanmar where mithun are naturally distributed [16]. Therefore, the knowledge from Dulongjiang area could be beneficial in the effective management of fodder resources and mithun, and hence it could also contribute to the livelihoods development in the region.

## Conclusion

The results of our research demonstrate the importance of traditional knowledge in identifying suitable tree fodder species. In addition, the diversity of species mentioned by respondents indicates that the study area is rich in forage plants which could be further investigated.

According to the information provided by our informants, fodder species such as *Erianthus longisetosus*, *Arundo donax* and *Paris* sp. may have interesting nutritional properties. However, at present scientific knowledge of these wild fodder species is too sparse to support the promotion of fodder trees at the farm level. Padmakumar et al. had listed nutritional value of some species closely related to our findings. This lead to the Ffurther research into these and other frequently-cited species which could help farmers select fodder trees which are ecologically suitable and economically beneficial [57]. Tree fodder species such as *Debregeasia orientalis* and *Saurauia polyneura* were highlighted by local farmers, as well as forage plants in the genus *Rubus*. Further studies are needed including a nutritional evaluation of various fodder species consumed by mithun and researches into the effects of different fodder species on rumen microbial flora and environment of mithun.
